# Epidemiology of Rugby-Related Injuries Presenting to the Emergency Department: A 10-Year Review

**DOI:** 10.7759/cureus.40589

**Published:** 2023-06-18

**Authors:** Haad Arif, Fatima Arif, Jose Morales, Ian W Waldrop, Nicholas W Sheets

**Affiliations:** 1 School of Medicine, University of California Riverside School of Medicine, Riverside, USA; 2 School of Medicine, Eastern Virginia Medical School, Norfolk, USA; 3 Trauma and Acute Care Surgery, Riverside Community Hospital, Riverside, USA

**Keywords:** covid-19, rugby, injury prevention, muscle injuries, medical aspects of sports

## Abstract

Background

Rugby is a popular contact sport played with little to no protective clothing. There exist few comprehensive studies investigating emergency department (ED) visit patterns for rugby-related injuries.We hypothesize that male athletes remain the most common patient demographic to present to the ED with rugby-related injuries and that the number of patients diagnosed with soft tissue injuries such as sprains and strains decreased during the COVID-19 pandemic.

Methodology

The National Electronic Injury Surveillance System database was examined for rugby injuries from January 2012 through December 2021. Cases were stratified by sex, age, and injury type to monitor epidemiological patterns. This is a descriptive epidemiology study. Level of evidence III.

Results

A total of 2,896 individuals with rugby-related ED visits were identified. ED patients were most common among males (73.9%), Caucasians (45.3%), and in the 15-19-year-old age range (44.9%). Injuries most commonly affected the upper body, specifically the head (23.1%), face (13.8%), and shoulder (12.4%) with fractures and sprains comprising 22.3% and 18.5% of ED diagnoses, respectively. Concussions were the most frequent injury to any one body part (11.2%). During the COVID-19 pandemic, ED patients with rugby-related injuries were significantly more likely to be males presenting with lacerations or hemorrhages. ED visits for sprains and strains significantly decreased in the peri-COVID-19 period.

Conclusions

Annual ED visits due to rugby injuries are declining. The head and neck are the most common sites of injuries. Decreased presentation to the ED during the COVID-19 pandemic may raise concern for the potential for untreated injuries. Physicians should anticipate the presence of chronic sports-related injuries when evaluating future patients.

## Introduction

Rugby is the ninth most popular sport worldwide, with 405 million fans worldwide, 45 million fans in the United States, 123 countries partaking in the sport, and many countries championing a national team [1-3]. According to USA Rugby, there are 1.4 million rugby players in the United States, 48% of whom are collegiate and high school players [3]. World Rugby estimates that over 9 million adults and children participated in the sport as of 2018, an increase of 5.9 million players from 2009. Given the lack of protective equipment used in rugby and its increasing popularity in the United States, a thorough understanding of injury patterns is the first step in the injury prevention sequence. Current work has examined the effects of implementing penalties in efforts to decrease risky gameplay and increase player safety [4]. Despite the wide range of player demographics, few studies have been done on a comprehensive review of rugby-related emergency department visits (RREVs) in the United States. Studies have either focused on specific injury sites such as the head, neck, or shoulder; compared injury rates of rugby to that of other contact sports such as American Football; or investigated the incidence of injuries at specific levels of gameplay (high school, college, or professional) [5-9]. These studies presented the following significant findings: (1) there is a dearth of research on the frequency of genitourinary injuries in rugby [5]; (2) the majority of rugby-related facial injuries are to the eyes or nose [6]; (3) shoulder injuries in rugby more commonly affect schoolboy athletes and are responsible for over one-third of all days absent [7]; (4) concussions, which account for a quarter of all days absent, may have been underreported in past years [8]; and (5) American football and rugby both pose similar risks of hospital admission [9].

The present study expands on the prior works to include the COVID-19 era when describing emergency department (ED) visits due to rugby-related injuries across all ages within the United States [10-12]. These studies found that the number of RREVs is increasing, with head and facial injuries accounting for one-third of injuries. All studies found male athletes to be most frequently injured, especially with head and facial injuries. Concussions were found to be more common in pediatric patients. By evaluating injury patterns, researchers may identify athlete demographics that are at an increased risk of specific injury types. Particularly, injury surveillance and investigation provide insights into position-specific, age-specific, and sex-specific injury risks that may be utilized in novel injury prevention strategies. We hypothesize that adolescent, male athletes would remain the most common demographic to be injured in rugby. We additionally believe that while RREVs overall would decrease during the COVID-19 lockdowns, visits due to soft tissue injuries such as contusions, sprains, and strains, in particular, would decrease significantly.

## Materials and methods

Data collection

The Consumer Product Safety Commission’s (CPSC) National Electronic Injury Surveillance System (NEISS) database is a nationally representative sample of 100 EDs throughout the United States. In NEISS hospitals, coders are trained to review all ED records daily, entering demographic, treatment, and injury details into the NEISS database and assigning each case with a CPSC-specific product code. This product code designates each case with the particular products or activities involved with the injury. Querying the database for a particular code will then return all ED visits that have been tagged with the corresponding code. Variables included in the database consist of treatment date, age, sex, race, injury occurrence location, diagnosis, body part injured, and disposition. Cases were divided into age groups of less than 10 years old, 10-14 years old, 15-19 years old, 20-24 years old, 25-29 years old, 30-34 years old, 35-39 years old, 40-44 years old, 45-49 years old, 50-54 years old, 55-59 years old, 60-64 years old, and 65-69 years old. Patient disposition was categorized as follows: treated and released, treated and transferred, admitted, held for observation, or left without being evaluated. Ethical approval was not required by the Institutional Review Board as the information is from a publicly available database and is de-identified.

The NEISS database was queried using the product code 3234 to identify rugby-related ED visits between January 2012 to December 2021 in patients of all ages. Narrative histories were reviewed to ensure appropriate coding. All ED visits where the patient sustained an injury due to participating in rugby were included in the data set. Narrative histories were independently reviewed by HA and JM. All cases in which the narrative description stated that the injury did not directly result from participation in rugby matches or practices were excluded. This exclusion criterion resulted in the removal of seven cases that mentioned rugby in passing but not as the cause of injury, leaving a total of 2,896 cases to be considered.

Statistical analysis

Data collection was performed using Microsoft Excel version 16.58 (Microsoft Corporation, 2022, Redmond, WA, USA). Bivariate comparisons between variables were conducted using chi-square analysis. P-values <0.05 (two-sided) were considered statistically significant.

## Results

Patient demographics

Our query of the NEISS database returned a total of 2,896 patients with relevant rugby-related injuries. Roughly 75% of patients were between the ages of 15 and 24 years old. Injury incidence initially increased with age, culminating with the 15-19-year-old age group, and then proceeded to decrease as patient age increased. Detailed demographic information is presented in Table 1.

**Table 1:** Demographics of rugby injury patients.

	N	%
Age range (years)		
<9	10	0.35%
10–14	196	6.77%
15–19	1,301	44.92%
20–24	865	29.87%
25–29	276	9.53%
30–34	145	5.01%
35–39	50	1.73%
40–44	21	0.73%
45–49	16	0.55%
50–54	8	0.28%
≥55	8	0.28%
Total	2,896	100.00%
Sex		
Male	2,141	73.93%
Female	755	26.07%
Total	2,896	100.00%
Race		
White	1,313	45.34%
African American	177	6.11%
Asian	46	1.59%
American Indian	7	0.24%
Native Hawaiian	37	1.28%
Unknown/Not stated	1,316	45.44%
Total	2,896	100.00%

Injury classification

The upper body was most commonly subject to injury with the head (23.1%), face (13.8%), and shoulder (12.4%) comprising a total of 49.3% of total injuries. The knee (9.5%) and ankle (8.2%) comprised the majority of injuries to the lower body. Injury diagnosis patterns showed sprains and strains comprising the largest portion (22.3%), followed by fractures (18.5%), and then lacerations (11.7%). The ankle (25.6%) and knee (21.4%) were the most likely sites to be sprained or strained. Fractures most commonly involved the face (22.6%) and the shoulder (17.6%). Lacerations most commonly affected the face (63.8%). Concussions comprised 11.2% of injuries. Greater than 90% of injuries occurred at a place of recreation or sports (80.2%) or school (11.1%). Most injuries were managed acutely in the ED or examined and discharged without treatment (96.1%), held for observation (0.5%), or left without being seen (0.8%). Our study found that only 2.6% of patients required hospital admission or transfer to another facility, typically due to fractures requiring surgery or brain injury (Table 2). A complete breakdown of injured body parts and diagnoses can be seen in Table 3.

**Table 2:** Diagnosis of admitted or transferred cases.

Diagnosis	Number of cases, N (%)
Lower extremity fracture, dislocation, or unspecified injury	24 (32%)
Upper extremity fracture, dislocation, or unspecified injury	13 (18%)
Concussion or unspecified closed head injury	11 (15%)
Facial injury, jaw fracture, nasal fracture, orbital fracture, ruptured globe	6 (8%)
Internal organ injury, splenic laceration, rectal bleeding, colon contusion, pancreatic laceration, renal hematoma	6 (8%)
Neurologic event, seizure, cerebral vascular accident, quadriparesis, temporal aneurysm, subarachnoid hemorrhage	5 (7%)
Thoracic injury, pneumothorax, pulmonary contusion, sternal fracture	4 (5%)
Miscellaneous, sepsis, rhabdomyolysis, compartment syndrome	3 (4%)
Neck fracture or dislocation	2 (3%)
Total	74

**Table 3:** Diagnosis by body part.

Diagnosis	Body part																
	Head	Face	Shoulder	Knee	Ankle	Trunk	Finger	Leg	Unknown	Neck	Hand	Wrist	Foot	Mouth	Arm	Elbow	Total
Sprain/Strain	-	-	124	138	165	37	35	23	1	49	11	32	12	-	5	13	645
Fracture	4	121	94	7	50	15	60	64	3	2	27	23	21	-	38	6	535
Other/Not stated	9	16	49	90	18	49	13	8	38	15	8	7	14	1	6	6	347
Laceration	42	208	-	8	-	-	2	6	15	-	2	-	1	41	-	1	326
Concussion	325	-	-	-	-	-	-	-	-	-	-	-	-	-	-	-	325
Internal injury	274	-	-	-	-	10	-	-	3	1	-	-	-	-	-	-	288
Contusion/Abrasion	13	43	28	15	2	71	7	18	14	2	18	3	12	2	3	11	262
Dislocation	-	1	64	17	1	5	31	1	-	-	1	1	-	-	-	10	132
Dental injury	-	-	-	-	-	-	-	-	-	-	-	-	-	12	-	-	12
Hematoma	1	2	-	-	-	2	1	1	2	-	-	-	-	-	-	-	9
Hemorrhage	-	8	-	-	-	-	-	-	-	-	-	-	-	-	-	-	8
Nerve damage	1	-	1	-	-	3	-	-	-	-	-	-	-	-	-	-	5
Crushing injury	-	-	-	-	1	-	-	1	-	-	-	-	-	-	-	-	2
Total	669	399	360	275	237	192	149	122	76	69	67	66	60	56	52	47	2,896

Injury by sex

The majority of injuries occurred in males (73.9%), who were most frequently injured in the face, shoulder, and mouth (p < 0.01). Women were most frequently injured in the head (p = 0.04), knee (p < 0.01), and ankle (p < 0.01) (Table 4).

**Table 4:** Sex differences in body parts injured.

Body Part	Male, N (%)	Female, N (%)	P-value
Head	474 (22.14%)	195 (25.83%)	0.0387
Face	338 (15.79%)	61 (8.08%)	<0.001
Shoulder	287 (13.40%)	73 (9.67%)	0.0075
Knee	167 (7.80%)	108 (14.30%)	<0.001
Ankle	158 (7.38%)	79 (10.46%)	0.0079
Trunk	142 (6.63%)	50 (6.62%)	0.9925
Finger	115 (5.37%)	34 (4.50%)	0.3533
Leg	97 (4.53%)	25 (3.31%)	0.1516
Neck	43 (2.01%)	26 (3.44%)	0.0262
Hand	48 (2.24%)	19 (2.52%)	0.6661
Wrist	43 (2.01%)	23 (3.05%)	0.1003
Foot	38 (1.77%)	22 (2.91%)	0.0589
Mouth	55 (2.57%)	1 (0.13%)	<0.001
Arm	42 (1.96%)	10 (1.32%)	0.2569
Elbow	32 (1.46%)	15 (1.99%)	0.3575
Other	62 (2.90%)	14 (1.85%)	0.1237
Total	2,141	755	

Men were more likely to be diagnosed with fractures (p < 0.01), lacerations (p < 0.01), and dislocations (p < 0.01). Women were more likely to be diagnosed with sprain/strains (p < 0.01), concussions (p < 0.01), and contusions (p < 0.01) (Table 5).

**Table 5:** Sex differences in diagnosis.

Diagnosis	Male, N (%)	Female, N (%)	P-value
Sprain/Strain	416 (19.43%)	229 (30.33%)	<0.001
Fracture	426 (19.90%)	109 (14.44%)	<0.001
Other/Not stated	251 (11.72%)	96 (12.72%)	0.471
Laceration	303 (14.15%)	23 (3.05%)	<0.001
Concussion	221 (10.32%)	104 (13.77%)	0.010
Internal organ injury	205 (9.57%)	83 (10.99%)	0.263
Contusion	174 (8.13%)	88 (11.66%)	0.004
Dislocation	113 (5.28%)	19 (2.52%)	0.002
Dental injury	12 (0.565%)	0 (0.00%)	0.040
Hematoma	8 (0.37%)	1 (0.13%)	0.306
Hemorrhage	7 (0.33%)	1 (0.13%)	0.382
Nerve damage	4 (0.19%)	1 (0.13%)	0.757
Crushing	1 (0.05%)	1 (0.13%)	0.441
Total	2,141	755	

Men were significantly more likely to be treated and admitted compared to women (p < 0.01). Women tended to be injured between 15-19 years of age (p < 0.01) while men were more likely to be injured between 30-34 (p = 0.04) and 35-39 years of age (p < 0.01).

Injury by age

Sprain/strain was the most common diagnosis in age groups of 15-19 years old (23.75%), 20-24 years old (21.73%), and 25-29 years old (21.01%). Fractures were the most common diagnosis in age groups of 10-14 years old (24.49%) and 35-39 years old (30%). Patients aged 30-34 years old were equally likely to suffer from a sprain/strain (22.07%) or a fracture (22.07%) (Table 6).

**Table 6:** Body part/diagnosis and age cross-tabulation.

Category		Age range											
		≤9	10-14	15-19	20-24	25-29	30-34	35-39	40-44	45-49	50-54	≥55	Total
Body part	Head	3	54	351	190	47	16	5	1	1	-	1	669
	Face	1	13	153	152	46	26	5	2	-	1	-	399
	Shoulder	1	18	168	109	37	14	5	2	5	1	-	360
	Knee	1	17	123	81	28	14	6	3	-	2	-	275
	Ankle	-	19	122	58	21	13	3	-	-	1	-	237
	Trunk	-	8	74	53	24	13	8	7	2	-	3	192
	Finger	-	16	66	34	17	8	4	1	1	1	1	149
	Leg	1	9	38	38	13	12	6	2	2	1	-	122
	Other/Not stated	1	6	32	24	7	5	-	-	-	-	1	76
	Neck	-	5	31	22	6	4	-	-	1	-	-	69
	Hand	-	7	22	22	7	4	4	-	1	-	-	67
	Wrist	-	6	32	17	5	4	1	-	-	-	1	66
	Foot	-	3	20	22	8	4	-	1	1	-	1	60
	Mouth	-	1	30	16	3	2	3	1	-	-	-	56
	Arm	2	9	20	13	3	2	-	-	2	1	-	52
	Elbow	-	5	20	14	3	4	-	1	-	-	-	47
Diagnosis	Sprain/Strain	1	35	309	188	58	32	7	4	6	3	2	645
	Fracture	2	48	223	154	52	32	15	3	5	1	-	535
	Other/Not stated	1	30	148	98	35	20	6	5	1	-	3	347
	Laceration	2	9	114	124	41	24	8	3	-	1	-	326
	Concussion	1	38	181	83	13	5	2	1	-	-	1	325
	Internal injury	2	11	142	91	29	10	1	-	2	-	-	288
	Contusion/Abrasion	1	20	119	69	28	13	5	3	1	1	2	262
	Dislocation	-	2	47	49	17	6	6	2	1	2	-	132
	Dental injury	-	-	10	2	-	-	-	-	-	-	-	12
	Hematoma	-	1	4	3	-	1	-	-	-	-	-	9
	Hemorrhage	-	1	4	3	-	-	-	-	-	-	-	8
	Nerve damage	-	-	1	1	1	2	-	-	-	-	-	5
	Crushing injury	-	1	-	-	1	-	-	-	-	-	-	2
	Total	10	196	1302	865	275	145	50	21	16	8	8	2896

Injuries by month and year

**Figure 1:**
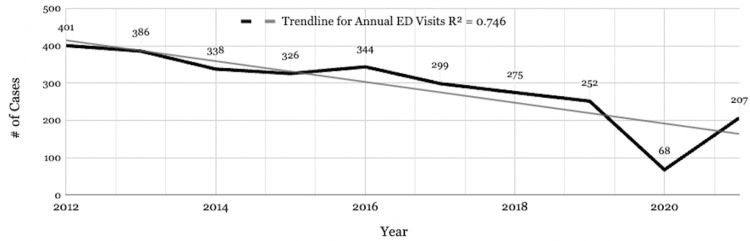
Total rugby-related ED visits by year. ED: emergency department

**Figure 2:**
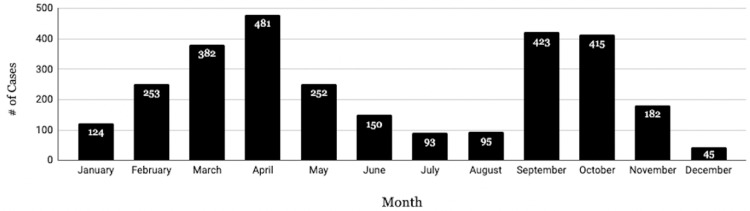
Total rugby-related ED visits. ED: emergency department

There is a downward trend in annual RREVs from 2012 (n = 401) to 2021 (n = 207) with a marked decrease in 2020 (n = 68) (Figure 1). Investigation of monthly ED visit patterns revealed a bimodal distribution across the 10 years, where RREVs spiked during the Spring (March and April) and Fall (September, October, and November) seasons, with the highest number of visits occurring in April for every year except 2020 (Figure 2).

Pre and peri-COVID-19 comparison

**Figure 3:**
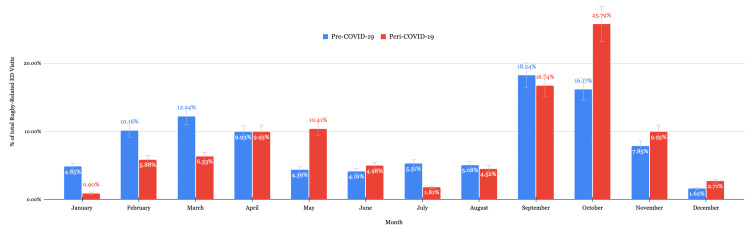
Comparison of monthly rugby-related ED visits. ED: emergency department; COVID-19: coronavirus disease 2019

Cases between 06/01/2018 and 03/11/2020 were considered pre-COVID-19. Cases after 03/11/2020 were defined as peri-COVID-19 data. RREVs decreased by 48.9% during the peri-COVID-19 era. During the peri-COVID-19 era, males were significantly more likely to present to the ED with rugby-related injuries while females were significantly less likely (p < 0.01 each). Injuries to the trunk significantly decreased in the peri-COVID-19 era (p < 0.01). Significant changes in diagnoses include increased laceration (p < 0.01), increased hemorrhages (p < 0.01), and decreased sprains and strains (p < 0.01) in the peri-COVID-19 era. ED visits significantly decreased in the peri-COVID-19 era during the months of January, March, and July, and significantly increased during the months of May and October (Figure 3). Detailed information can be found in Table 7. Schoolyard injuries also decreased during the COVID-19 pandemic (p = 0.03). There was no significant difference in the age of patients.

**Table 7:** Pre-COVID-19 versus peri-COVID-19 ED visit comparison. ED: emergency department; COVID-19: coronavirus disease 2019

	Category	Pre-COVID-19, N (%)	Peri-COVID-19 N (%)	P-value
Age range	≤15	65 (15.01%)	30 (13.57%)	0.622
	16-20	199 (45.96%)	107 (48.42%)	0.551
	21-25	86 (19.86%)	57 (25.79%)	0.080
	26-30	42 (9.70%)	12 (5.43%)	0.060
	>30	42 (9.70%)	15 (6.79%)	0.212
	Total	433	221	
Sex	Male	308 (71.13%)	180 (81.45%)	0.004
	Female	125 (28.87%)	41 (18.55%)	0.004
	Total	433	221	
Injury site	Head	97 (19.32%)	55 (20.15%)	0.704
	Face	62 (12.35%)	38 (13.92%)	0.480
	Knee	55 (10.96%)	32 (11.72%)	0.691
	Shoulder	45 (8.96%)	29 (10.62%)	0.411
	Ankle	44 (8.76%)	20 (7.33%)	0.523
	Hand	35 (6.97%)	19 (6.96%)	0.231
	Leg	33 (6.57%)	17 (6.23%)	0.924
	Trunk	32 (6.37%)	8 (2.93%)	0.031
	Other	99 (19.72%)	55 (20.15%)	0.887
	Total	502	273	
Diagnosis	Sprain/Strain	104 (21.76%)	32 (12.50%)	0.002
	Fracture	98 (20.50%)	52 (20.31%)	0.952
	Laceration	34 (7.11%)	37 (14.45%)	0.001
	Concussion	50 (10.46%)	23 (8.98%)	0.524
	Internal injury	37 (7.74%)	26 (10.16%)	0.265
	Dislocation	15 (3.14%)	15 (5.86%)	0.076
	Hemorrhage	0 (0.00%)	5 (1.95%)	0.002
	Other	140 (29.29%)	66 (25.78%)	0.722
	Total	478	256	

## Discussion

RREV and injury site

Our review of the literature found three prior studies using the NEISS database which reported RREVs in the United States from 1978 to 2004, 2004 to 2013, and from 1999 to 2018 [10-12]. These studies have found that sprains, lacerations, fractures, and contusions have historically been the most common injuries, with males being more susceptible to facial injuries, lacerations, and dislocations while females more often suffered knee injuries and contusions [11,12]. Our study found similar results, with sprains, fractures, and lacerations being the top three injury diagnoses across the entire body. However, we found that concussions comprised roughly 11% of ED diagnoses, emphasizing the relative concentration of internal head injuries when compared to diagnoses spread out across all body parts such as bony injuries, contusions, or lacerations. Studies have shown that the majority of injuries, regardless of athlete sex, are sustained during tackling situations, which rugby coaches have attempted to combat by emphasizing proper tackling technique at a young age [6,13-15]. Injury prevention programs have previously focused on reducing the injury burden related to the scrum, which may be a causative factor in the shift to open-field, tackle-related injuries [16]. In both adolescent and professional gameplay, the tackler faces a higher risk of injury compared to the ball carrier [16]. While proper tackling techniques are designed to place the head, face, and neck out of harm’s way, they increase the likelihood of shoulder injury by placing a greater load on the shoulder. This is consistent with our study which revealed the shoulder is the second most common site of fractures in males and females alike. A previous study in 2021 found an even higher incidence of shoulder fractures than the present investigation, stating that 40.5% of fractures occurred in the shoulder [10,14]. Protective shoulder padding may serve to decrease the incidence of tackle-related injuries. Similar to Kim et al., we found no significant injury burden to the trunk or pubic area [5].

Sex differences in RREVs

The prevalence of injuries in male athletes has been well documented in the literature. Indeed, our study showed that males made up 73.9% of RREVs, which is comparable to a prior study in 2021 that found male patients to comprise 79.3% of RREVs [10]. This may be due to the prevalence of male athletes in rugby as opposed to a true difference in injury rate between sexes. Studies have stated that male players have more overall injuries and are injured more often during matches, while females are more predisposed to anterior cruciate ligament injuries and are more often injured during training [17,18]. One study found that while both males and females suffered similar rates of head, neck, and face injuries (HNFIs), the cause of injury differed between the two sexes [19]. Males sustained HNFIs mostly due to contact with another player while females sustained HNFIs due to impact with the playing surface [19]. We found males more likely to sustain more severe head and shoulder injuries (fracture, dislocation, and laceration) requiring hospital admission as compared to females who sustained injuries to the lower extremities (knee and ankle) that may be considered to be less severe (sprain/strain and contusion). Proposed theories behind sex differences in rugby injuries include a higher frequency of foul play in men’s games or an increased likelihood of a background playing American football in male players, both of which would result in poor adherence to safe play practices emphasized by rugby guidelines and may explain both the increased incidence of male ED visits found in our study as well as the differences in the mechanism of HNFI found by Ma et al. [9,19,20]. Increased muscle mass and velocity of play may be additional reasons for the prevalence of the higher number of male patients in RREVs. Our study found the head and face to be the most common sites of rugby-related injury among both sexes. Sabesan et al. suggested mandated usage of a scrum cap; however, further prospective research is required to understand whether a scrum cap may truly mitigate concussion risk [11].

RREVs and age

Our study’s finding of increased injury incidence with age is in line with many studies done previously, which found that young adult players typically have more injuries than their adolescent counterparts [11,15]. This may be due to the increased confidence in athletic ability, increased muscle mass, and fast speed of play that comes with increased age resulting in a higher risk of injury. As age surpasses young adulthood, athletes may tend to practice a more reserved and technical playing style and play with decreased aggression due to cognizance of their increasing fragility. All of these factors could contribute to the downtrend in injuries seen in our results from 20 to 69 years old. Decreased participation in rugby at an advanced age may also contribute to decreased rates of RREV. We found concussions to be most common in patients under the age of 15 years, further validating the increased emphasis on teaching safe tackling techniques at an early age.

Injury burden is generally higher at professional levels of play with up to a 34% higher injury incidence in professional rugby athletes compared to amateur and recreational athletes [16]. This has been postulated to be due to increased competitiveness and intensity, a greater number of collisions, and longer seasons [11,15,16]. Some studies have also considered the lack of injury reporting in amateur-level gameplay due to the decreased presence of medical personnel [7].

RREVs throughout the year

There is a clear bimodal monthly pattern of ED visits due to rugby-related injuries, with visits spiking in April and then in September. Rugby is traditionally a Fall sport, beginning in September in many countries; however, there are multiple international leagues and tournaments with no standardized world rugby calendar [13,15,21]. The professional clubs of Major League Rugby play their season from March to July, while the amateur club rugby season in the United States spans from September to May and is played in high schools, colleges, and universities [21]. School-age players comprise the majority of rugby athletes in the United States [8]. The surge of spring-time rugby injuries in our study may be due to the overlap between amateur and professional rugby seasons during March, April, and May. Reported injuries drop during July and August as the seasons come to a close before spiking again in September. With the majority of players participating in the Fall season being high-school and college students, injury incidence may decrease in conjunction with students’ academic calendars/final exam schedules and Christmas holiday break.

RREVs and COVID-19

The COVID-19 pandemic introduced widespread concern regarding player safety, with many professional sporting organizations implementing player suspensions for positive COVID-19 tests [22]. While ED visits for injuries sustained due to participation in many organized team sports including ice hockey, American football, baseball, softball, and basketball decreased by 53.9% during the COVID-19 pandemic, injury incidence for various sports has been shown to have increased. Puga et al. found a statistically significant increase in the prevalence of athletic injuries in the National Football League during the COVID-19 pandemic, which suggests decreased physiological adaptation to stress, due to the limited amount of training as a result of the closure of practice facilities [23]. Our findings show that RREVs also demonstrated a similar decrease of 48.9% between the pre- and peri-COVID-19 periods; however, given that injury incidence in other contact sports has been shown to have increased, this decrease in RREVs may not be representative of a decrease in absolute injury occurrence. The overall annual decrease in RREVs during the COVID-19 pandemic could be attributed to the government-mandated lockdowns, a transition to virtual classrooms and online education, and widespread public health campaigns advising against close person-to-person contact. Injured athletes may have also not gone to the ED during this time out of fear of the hospital environment and contracting the virus, especially for seemingly minor injuries such as sprains and strains. This may subsequently bias our results to patients presenting with more severe injuries. Males have historically been more susceptible to more severe injuries that may prompt ED presentation while females are more likely to sustain superficial injuries such as contusions and lacerations [19]. This may help to explain our findings of a statistically significant decrease in female patients and patients diagnosed with strains or sprains.

The increased usage of telemedicine appointments during the COVID-19 pandemic may have helped mitigate the aversion to seeking medical care; however, if this truly is the case, these findings present cause for concern due to the prevalence of untreated sports-related injuries that may have long-term implications for patient health and safety. Therefore, physicians should maintain a high degree of suspicion for underlying, chronic injuries when evaluating patients presenting for rugby-related injuries and practice a high threshold of treatment and recommendation of time away from play. This recommendation is especially important when evaluating for concussions due to their benign presentation (nausea, headache) that may go unrecognized by the general population.

As federal government regulations were lifted in 2021, some contact sports experienced increased sporting-related injuries to above pre-pandemic levels [24,25]. Tak et al. discuss that this may be due to athletes being out of practice for extended periods as a result of missed practices and matches [25]. While RREVs certainly increased in 2021 when compared to 2020, the downward trend of annual RREVs during this study period continued into the year 2021, keeping ED visits below pre-pandemic levels. This may be due to ongoing limitations and restrictions to gameplay that were kept in place by individual organizations and institutions.

Increasing player safety

Understanding factors accounting for the higher incidence of injury among male rugby players, susceptibility to injury based on age group, and injury frequency by the time of year could provide valuable insights into not only the prevention of injury but also streamlined management of injuries once patients present to the ED. Use of this understanding has already been employed by prominent rugby organizations, with advancements in literature directing new approaches to injury prevention. World Rugby has implemented numerous new safety protocols since 2019, such as limiting practices to 15 minutes of full contact, the optional use of goggles during gameplay, and the Activate Injury Prevention Program. The Activate Injury Prevention Program is a rugby-specific exercise program consisting of position, mobility, and strengthening exercises targeting the lower limb, shoulder, head, and neck, aimed at reducing rugby injuries [26]. The Activate Injury Prevention Program has since been accessed by over 30,000 individuals in 172 countries, demonstrating a 40% decrease in soft tissue injuries and up to a 60% reduction in concussion incidence [3,27]. The High Tackle Sanction Framework is another concussion mitigation program that highlighted decreasing high-risk upright impacts while increasing the lower-risk bent-at-the-waist tackling techniques [3]. In July 2021, World Rugby funded research and implemented the use of smart biometric mouthguards to gain a better understanding of concussions and the mechanism of injury [27]. Finally, new welfare-focused laws and regulations have been added to the official rugby rulebook since 2021, instituting penalties for risky player behavior such as targeting a player’s lower limbs while contesting for the ball [27]. While this study did not specifically investigate injury incidence, all of these strategies may have played a role in the declining rates of ED visits due to rugby-related injuries that we observed.

Limitations

There are several limitations of this study. While the NEISS provides a good sample representation of injury incidence, it is not feasible for all rugby-related injuries to be reported as it does not cover primary care clinics, urgent care clinics, orthopedic clinics, personal/athletic trainers, or individuals who did not seek medical attention. Thus, the NEISS database cannot effectively capture low-acuity injuries that did not warrant presentation to the ED. The sampling bias can also include geographical discrepancies, although the NEISS EDs are strategically selected to mitigate this bias. Because product codes are assigned by coders, there is also the possibility of errors in coding or data entry. Finally, as the NEISS database does not record national participation in sports, the use of this data cannot evaluate the total injury rates due to participation in rugby.

## Conclusions

From 2012 to 2021, the majority of patients presenting to the ED for rugby-related injuries consisted of males, Caucasians, and 15-19-year-old athletes diagnosed with sprains/strains, fractures, and lacerations. The head and neck remain the most common sites of injuries among all players, followed by the shoulder in men and the knee in women.

Across the study period, the number of rugby-related ED visits declined, especially in the recent COVID-19 era. While specific ED visit patterns have not changed drastically over the past decade, the decrease in ED visits highlights the successes of rugby organizations, coaches, and medical professionals in mitigating injury risk. Concussions comprise a substantial proportion of rugby-related ED visits, emphasizing the ongoing need to improve concussion prevention and monitoring in rugby. There was a significant increase in male patients and a significant decrease in soft tissue injuries during the COVID-19 pandemic. Given the potential for untreated sports injuries during the COVID-19 pandemic, clinicians should anticipate the likelihood of previous or existing sports-related injuries when evaluating future patients. Further prospective research is warranted to identify the impact of padded jerseys and scrum caps on decreasing rugby-related ED visits.
